# Fractures of the appendicular skeleton and criteria for their surgical management: pictorial essay

**DOI:** 10.1590/0100-3984.2021.0039

**Published:** 2022

**Authors:** Lucas Kenzo Miyahara, Letícia dos Reis Morimoto, Victor Cavalcante Schussel, Maurício Pandini Monteiro de Barros, Adham do Amaral e Castro

**Affiliations:** 1 Escola Paulista de Medicina da Universidade Federal de São Paulo (EPM-Unifesp), São Paulo, SP, Brasil.

**Keywords:** Fractures, bone/diagnostic imaging, Fractures, bone/surgery, Skeleton/injuries, Bone and bones/injuries, Joints/injuries, Fraturas ósseas/diagnóstico por imagem, Fraturas ósseas/cirurgia, Esqueleto/lesões, Osso e ossos/lesões, Articulações/lesões

## Abstract

The high prevalence of fractures and the essential role that imaging examinations play in this scenario require the radiologist to be familiar with their main patterns, especially those of fractures for which the management is essentially surgical. This pictorial essay presents a series of illustrative cases and a brief review of the literature, the aim being to demonstrate some of the main fractures of the appendicular skeleton that require surgical management, grouped, didactically, by the joint affected. Radiographic and computed tomography studies of illustrative cases were selected from the imaging archives of our facility.

## Abstract (pt)

A elevada prevalência de fraturas e o importante papel dos exames de imagem nesse contexto requerem que o radiologista esteja familiarizado com seus principais padrões, especialmente as fraturas com manejo essencialmente cirúrgico. Este estudo apresenta uma série de casos ilustrativos e uma breve revisão da literatura, com o objetivo de demonstrar algumas das principais fraturas do esqueleto apendicular com manejo cirúrgico, agrupadas didaticamente por articulação. Foram selecionadas radiografias e tomografias computadorizadas de casos didáticos ilustrativos do arquivo de imagens do nosso serviço.

## INTRODUCTION

Imaging examinations performed for the diagnosis of fractures due to trauma are highly prevalent in radiology practice, playing an essential role because they facilitate not only the diagnosis but also the classification of the injuries. The imaging examinations usually performed in this context are conventional radiography and computed tomography (CT). The former is used as an initial imaging method, because it demonstrates bone structures well, is widely available, and is affordable, whereas the latter allows three-dimensional reconstructions, visualization of structures in other planes (providing a more satisfactory assessment of complex fractures), and better evaluation of soft tissues, such as blood vessels. The evaluation of these imaging examinations is sometimes a challenging task, especially in patients with fractures for which the management is essentially surgical, in which a delayed diagnosis can have catastrophic consequences, as is the case for the various types of fractures addressed in this study. Therefore, it is essential to recognize the main patterns of fractures and their mechanisms of trauma, as well as imaging findings that in themselves denote a greater need for surgical treatment^(1)^.

This pictorial essay presents illustrative cases of the main fractures of the appendicular skeleton that require surgical management, grouped, didactically, by the joint affected. The focus is on X-ray and CT, the two methods most commonly used in emergency settings.

## UPPER LIMBS

### Proximal humerus fracture

**Figure 1:**
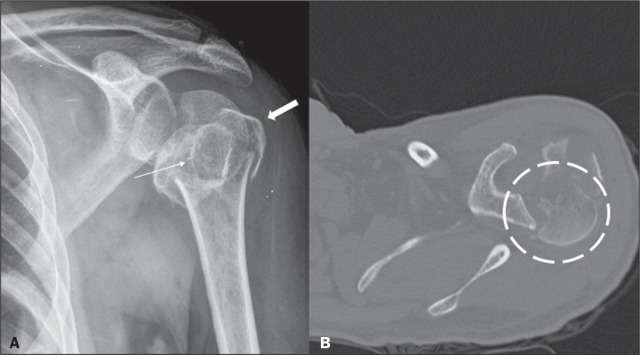
A 59-year-old man who had fallen seven days prior, evolving to pain in the left shoulder girdle region. Anteroposterior X-ray of the left shoulder (A) and CT of the left shoulder in the axial plane and in the bone window (B) demonstrate a comminuted metaepiphyseal fracture of the proximal humerus (Neer four-part fracture), showing the involvement of its surgical and anatomical neck; greater and smaller tuberosities (thick and thin arrows, respectively), with posteromedial dislocation of the humeral head (dashed circle) and extension of the fracture line to the glenohumeral joint.

Fracture of the proximal humerus is one of the most common fractures of the appendicular skeleton, accounting for 4.0-5.7% of all cases, and can occur in low- or high-energy trauma, such as falls in the elderly and motor vehicle accidents in young people. The Neer classification is used in order to assess and determine guidelines for the treatment of these fractures, classified as one to four parts: humeral head; greater tuberosity; lesser tuberosity; and diaphysis. Deviation between the parts is defined as when the distance between two parts is > 1.0 cm or the angle is > 45°^(1,2)^, as illustrated in Figure 1.

### Terrible triad of the elbow

**Figure 2:**
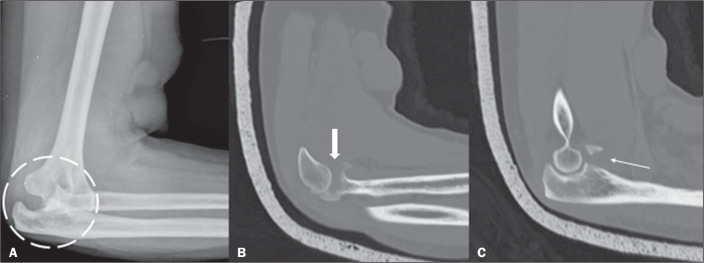
A 62-year-old woman who had sustained a trauma to the right upper limb, resulting from a fall from standing height with the elbow extended. Lateral X-ray of the elbow (A) showing posterior dislocation (dashed circle), and CT scan obtained with a plaster cast after closed reduction (B,C) showing a comminuted fracture of the radial head (thick arrow) and fracture of the coronoid process (thin arrow).

Characterized by posterior dislocation of the elbow together with a fracture of the coronoid process and radial head, the terrible triad of the elbow typically results from a fall onto an outstretched hand. It is usually accompanied by extensive ligament injury and, if inadequately treated, can progress to chronic instability and osteoarthritis^(1,3)^. Therefore, in patients with posterior dislocation of the elbow and fracture of the radial head, a CT study is recommended as a means of obtaining a more accurate diagnostic accuracy (Figure 2). Treatment of the terrible triad of the elbow is essentially surgical, the aim being to restore the congruence and stability of the joint^(1,3)^.

### Monteggia fracture-dislocation

**Figure 3:**
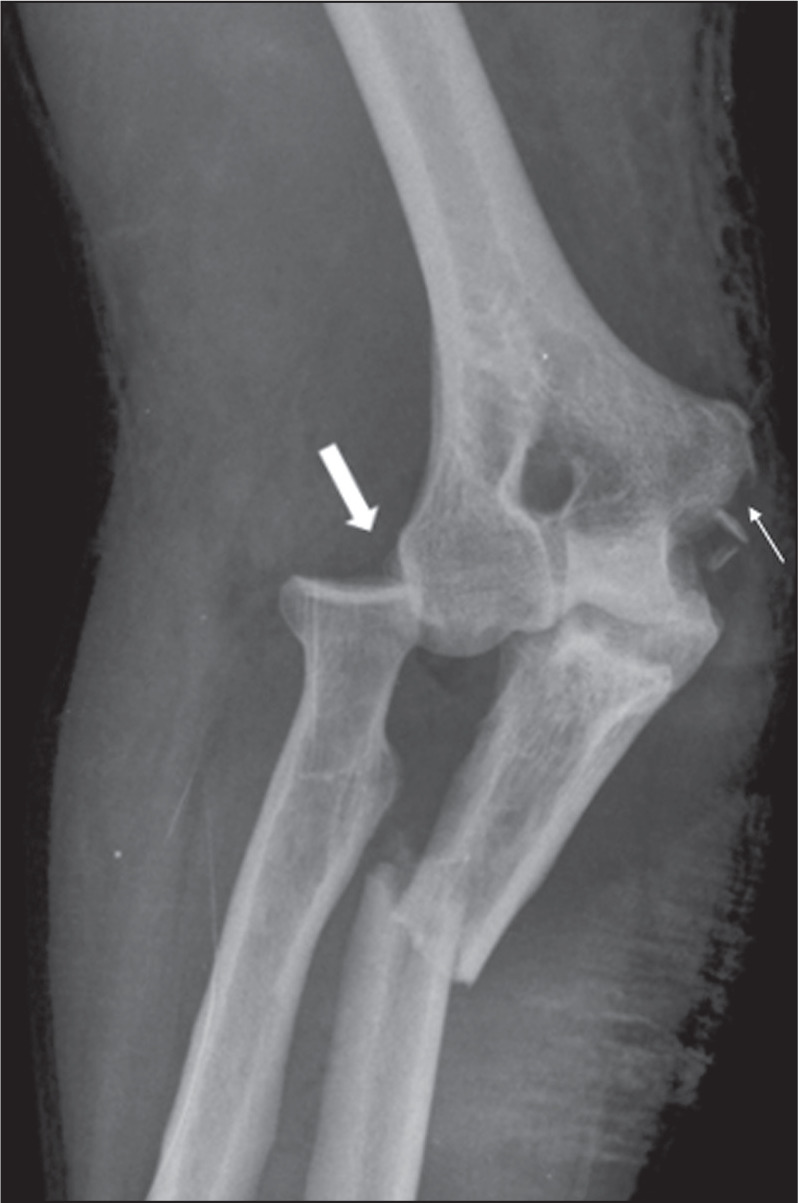
A 44-year-old man who had fallen from an overhead duct onto his right forearm, evolving to pain and deformity. Anteroposterior X-ray of the right elbow showing a Bado type III Monteggia fracture-dislocation, characterized by a fracture in the proximal third of the ulna with lateral dislocation of the radial head (thick arrow). Note also the trace fracture in the medial epicondyle (thin arrow), due to avulsion.

A Monteggia fracture-dislocation consists of a fracture of the ulnar diaphysis with dislocation of the radial head, the main mechanism of trauma being a fall onto an outstretched hand. This type of fracture-dislocation is more common in children, with a peak incidence between 4 and 10 years of age. The Bado classification subdivides Monteggia fracture-dislocations into four types, by the direction of displacement of the radial head: type I, when there is a fracture of the diaphysis of the ulna, with anterior angulation of the fracture focus and anterior dislocation of the radial head; type II, when there is a fracture of the diaphysis of the ulna, with posterior angulation of the fracture focus and posterolateral dislocation of the radial head; type III, when there is a fracture of the ulnar metaphysis with lateral or anterolateral dislocation of the radial head (Figure 3); and type IV, when there is a fracture of the proximal third of the radius and ulna, with anterior dislocation of the radial head. In the radiographic evaluation, fracture of the diaphysis of the ulna is usually easily recognized, making it necessary to investigate the dislocation of the radial head^(1,3)^.

### Galeazzi fracture-dislocation

**Figure 4:**
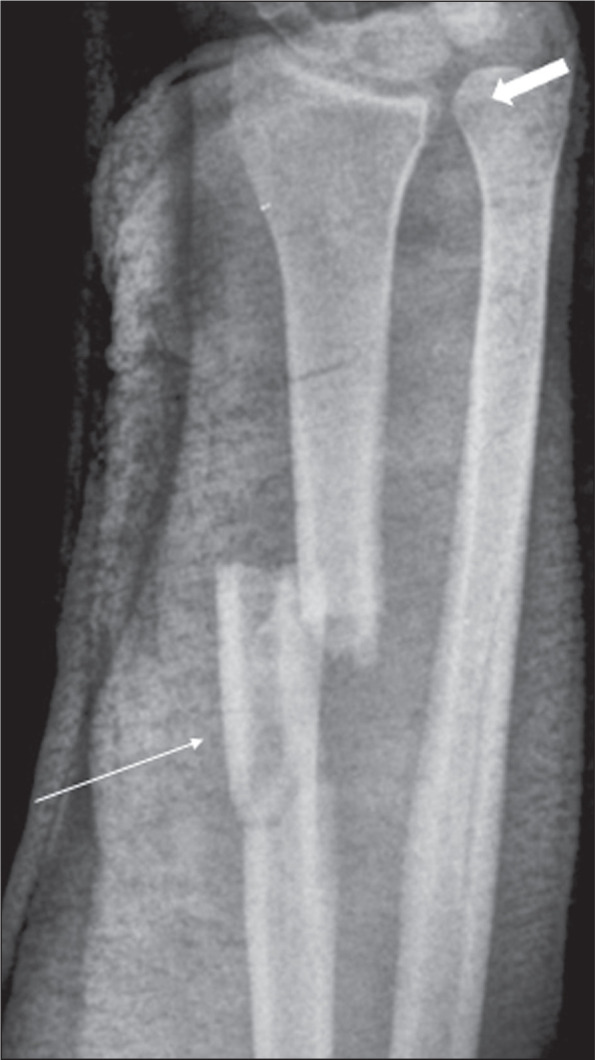
A 27-year-old man who had been struck by a motor vehicle traveling at high speed. Anteroposterior X-ray of the forearm taken with a plaster splint showing a fracture of the distal radial diaphysis (thin arrow), accompanied by diastasis of the distal radioulnar joint (thick arrow) and shortening of the radius.

Often misdiagnosed, a Galeazzi fracture-dislocation, previously known as a “fracture of necessity”, is a fracture of the distal diaphysis of the radius, together with injury to the distal radioulnar joint. Galeazzi fracture-dislocations account for less than 3% of forearm fractures in children and less than 7% of those in adults, typically resulting from a fall onto an outstretched hand with the elbow flexed. Assessment of the distal radioulnar joint is essential. As illustrated in Figure 4, the signs of a Galeazzi fracture-dislocation on X-ray include the following^(1,4)^: fracture of the ulnar styloid process; diastasis of the distal radioulnar joint on an anteroposterior X-ray; shortening of the radius to ≥ 5.0 mm; and volar or dorsal displacement on a lateral X-ray.

### Barton’s fracture

**Figure 5:**
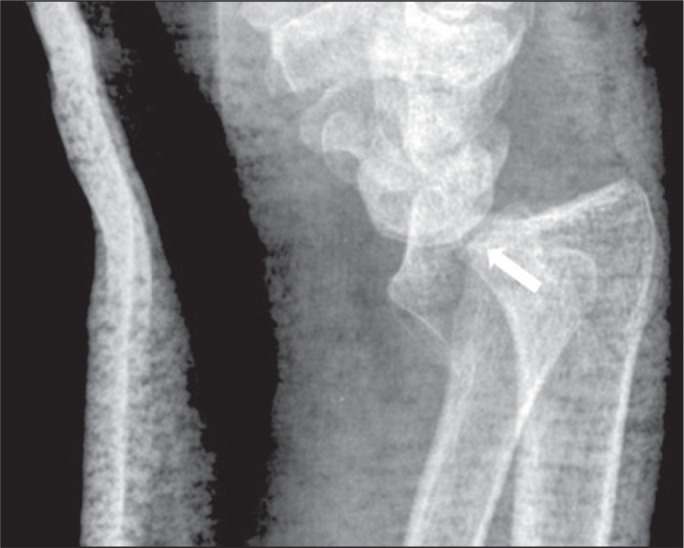
A 69-year-old man who had suffered an accident with a sander. Lateral X-ray of the wrist showing a volar Barton fracture, characterized by a partial fracture of the radius, extending to the joint (arrow), together with volar dislocation of the carpus and loss of radiocarpal alignment.

Defined as partial articular fracture of the distal radius in the sagittal plane, Barton’s fracture is more common in women and has a bimodal distribution, resulting from high-energy trauma in young people and falls in the elderly. Its management is essentially surgical. A Barton’s fracture can be categorized as volar or dorsal, depending on the direction of the deviated fragment^(1,5)^, as depicted in Figure 5.

### Scaphoid fracture

**Figure 6:**
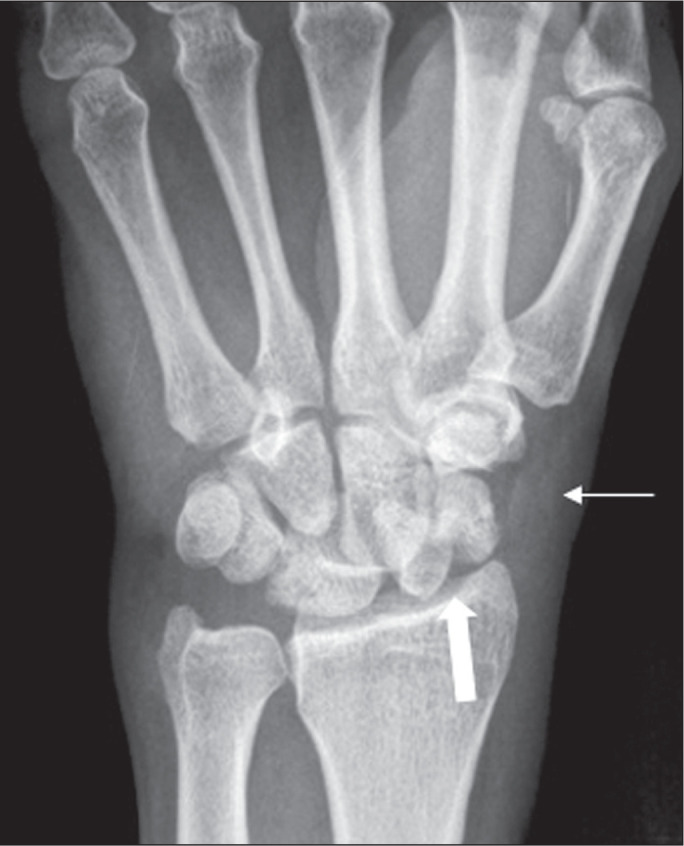
A 53-year-old man who had fallen from a height of 2 m, evolving to pain, edema, and restricted movement in the left wrist. Anteroposterior X-ray of the wrist showing a fracture of the scaphoid neck (thick arrow), in addition to densification and edema of the soft tissue on the lateral face of the wrist, with obliteration of the scaphoid fat stripe (arrow).

A scaphoid fracture (Figure 6) is the most common fracture of the carpus, occurring predominantly in active men, with peak incidence in the second and third decades of life, mostly resulting from a fall onto an outstretched hand with forced extension of the wrist. Delayed diagnosis of a scaphoid fracture is a common problem, and inappropriate treatment can result in complications, including pseudarthrosis and avascular necrosis^(1,6)^.

The findings, classifications, and indications for the surgical management of the fractures of the upper limbs described in this essay are summarized in Table 1.

**Table 1:** Upper limb fractures and their respective mechanisms of trauma, findings that indicate surgical treatment, and the main surgical treatment adopted.

Fracture	Segment	Mechanism of trauma	Findings that indicate surgical treatment	Main surgical treatment adopted
Proximal humerus	Proximal humerus	Elderly: falls; young people: high energy traumas	Marked varus or valgus displacement; deviated fracture of the greater or lesser tuberosity; fracture- dislocation; humeral head splitting	Open reduction and internal fixation with a plate and a screw; closed percutaneous reduction and fixation with wires or screws; intramedullary nailing; arthroplasty
Elbow (terrible triad of the elbow)	Radius and proximal ulna	Fall onto an outstretched hand	Loss of congruence of the humeroulnar and humeroradial joints; head fracture with pronation- supination blockage; fracture > 50% of the height of the coronoid process	External fixation; open reduction with internal fixation; ligament repair; radial head excision; radial head arthroplasty
Forearm (Monteggia fracture-dislocation, Galeazzi fracture- dislocation)	Radius and ulna diaphysis	Fall onto an outstretched hand	Monteggia or Galeazzi fracture-dislocations are essentially surgical cases, unless clinical conditions preclude surgery. Signs of injury to the distal radioulnar joint: fracture of the ulnar styloid process; diastasis of the distal radioulnar joint on an anteroposterior X-ray; radial shortening ≥ 5.0 mm; volar or dorsal displacement on a lateral X-ray. Signs of proximal radioulnar joint injury: break in the radiocapitellar line	Open reduction with internal fixation of the fractured bone and assessment of the stability of the joint affected
Distal radius (Barton’s fracture)	Distal radius	Fall onto an outstretched hand	Barton fractures are eminently surgical due to the instability and shear of the fragment. Other injuries: joint depression > 2.0 mm; radial shortening > 3.0 mm; dorsal angulation > 10° or > 20° in relation to the contralateral side. Associated injury (e.g., to a ligament or the ulnar styloid)	Open reduction with internal fixation; external fixation; closed reduction and percutaneous fixation
Scaphoid	Carpus	Fall onto an outstretched hand	Proximal pole fracture: displacement > 1.0 mm; intrascaphoid angle > 35°; comminuted fracture; carpal instability	Percutaneous fixation; open reduction with internal fixation; arthroscopy- assisted reduction and fixation

## LOWER LIMBS

### Pelvic ring fracture

**Figure 7:**
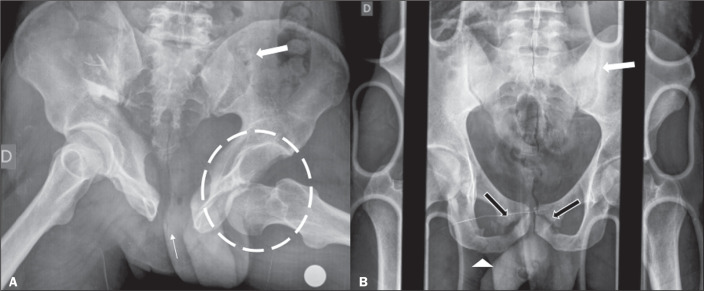
A: Anteroposterior X-ray of the pelvis of a 39-year-old man who had sustained an axial trauma due to a fall of approximately 3 m, showing marked diastasis of the pubic symphysis (thin arrow), diastasis of the left sacroiliac joint (thick arrow) and anterior dislocation of the left hip (dashed circle). B: Anteroposterior X-ray of the pelvis of a 25-year-old man, victim of a motorcycle-versus-car trauma, showing fractures of the right ischiopubic ramus (arrowhead) and bilateral pubic bone (black arrows), together with diastasis of the left sacroiliac joint (white arrow).

Typically, pelvic ring fractures result from high-energy blunt trauma, such as motor vehicle accidents, and are associated with high mortality rates (up to 50% for open fractures), hemorrhage being the main cause of death. The radiological evaluation begins with the acquisition of X-rays in anteroposterior, oblique, inlet and outlet views (Figure 7). Complementation with CT is routinely performed, special attention being paid to the integrity of the pubic symphysis, sacroiliac joints, pubic branches, iliac bone, and sacrum^(1,7)^.

### Intertrochanteric fracture

**Figure 8:**
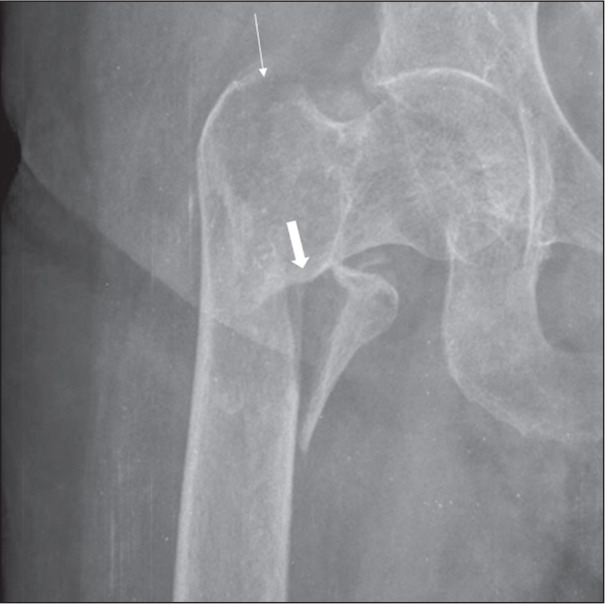
A 63-year-old woman who had fallen out of bed. Anteroposterior X-ray of the right hip showing a marked reduction in bone density with a complete fracture (thin arrow) affecting the greater and lesser trochanters. Note the involvement of the posteromedial cortex (thick arrow), resulting in fracture instability.

Intertrochanteric fractures are extracapsular fractures involving the greater or lesser trochanter (Figure 8).Because they are closely associated with osteoporosis, their incidence is higher in the elderly and in women. They usually occur after a fall in which there is a lateral impact on the greater trochanter. Intertrochanteric fractures are associated with high mortality, with a one-year mortality rate of up to 30%; however, surgery in conjunction with early rehabilitation reduces the associated morbidity and mortality^(1,8)^.

### Femoral neck fracture

**Figure 9:**
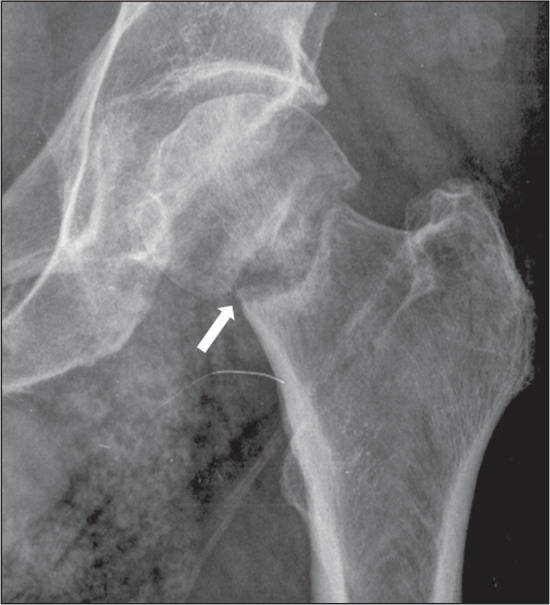
An 83-year-old man who had fallen from standing height. Anteroposterior X-ray of the left hip showing a fracture of the femoral neck (arrow).The trabeculae of the femoral head and acetabulum are parallel, characteristic of a complete and fully displaced fracture of the femoral neck.

Femoral neck fracture is most common in the elderly and in females. Such fractures are associated with a high mortality rate, the one-year mortality rate being approximately 25%. The mechanism of trauma in femoral neck fracture depends on the age and functional status of the patient, being a low-energy lateral fall with impact on the greater trochanter in older patients and high-energy trauma in younger patients. The most widely used classification is the Garden classification, which describes four categories of femoral neck fracture: incomplete or valgus impacted (type I); complete and nondisplaced (type II); complete and partially displaced (type III); and complete and fully displaced (type IV), as shown in Figure 9. Due to the retrograde irrigation of the femoral head, two complications are feared: osteonecrosis and pseudarthrosis^(1,8)^.

### Tibial plateau fracture

**Figure 10:**
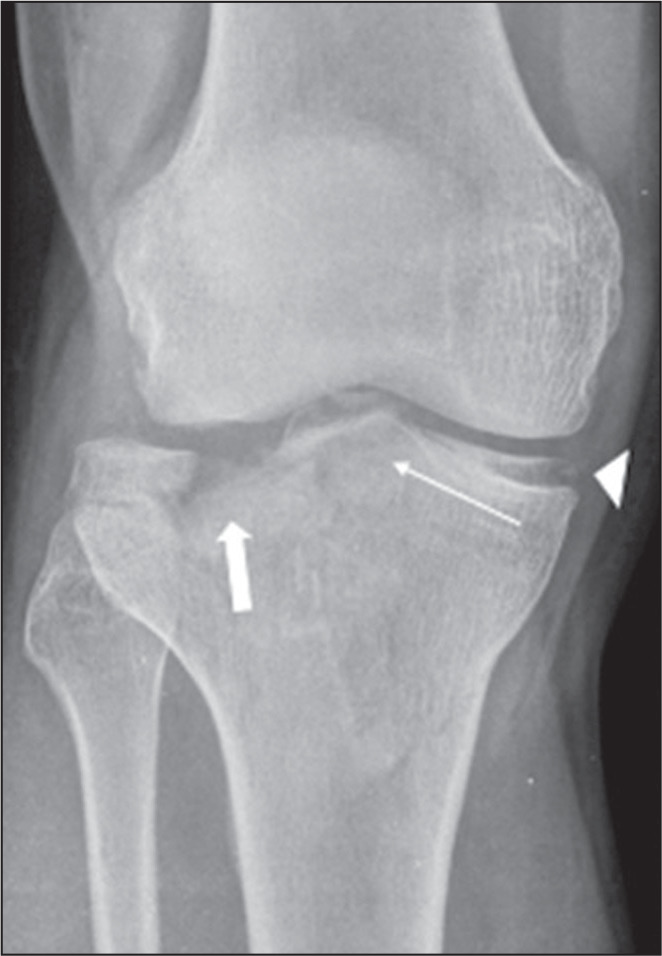
A 36-year-old man, victim of being struck by a bicycle, who evolved to pain in the right knee. Anteroposterior X-ray of the right knee showing a comminuted bicondylar fracture with significant depression of the lateral condyle of the tibia (thick arrow) and involvement of the tibial spines (thin arrow). Fracture of the medial tibial condyle, characterized by the double line (arrowhead), which, in and of itself, indicates greater severity of the injury, because it represents the load area of the joint.

Tibial plateau fractures are joint fractures of the proximal tibia that are typically accompanied by injuries to soft tissues such as ligaments and menisci. The most common mechanism is trauma with an axial force vector, such as that caused by a fall from a great height. The classification system that has long been used is the Schatzker classification, which divides tibial plateau fractures into six types, depending on the condyle affected and the presence or absence of joint depression, shear, or both. The first three types are pure tibial plateau fractures, typically associated with low-energy trauma: shear fracture of the lateral plateau fracture without depression (type I); shear and depression of the lateral plateau (Type II); and isolated depression of the lateral plateau (Type III).The remaining three types are more severe and associated with significant soft tissue damage^(1,9)^ : shear, depression, or both of the medial plateau (Type IV); bicondylar plateau fracture (Type V), as depicted in Figure 10; and complete dissociation between the metaphysis and diaphysis (Type VI).

### Ankle fracture

**Figure 11:**
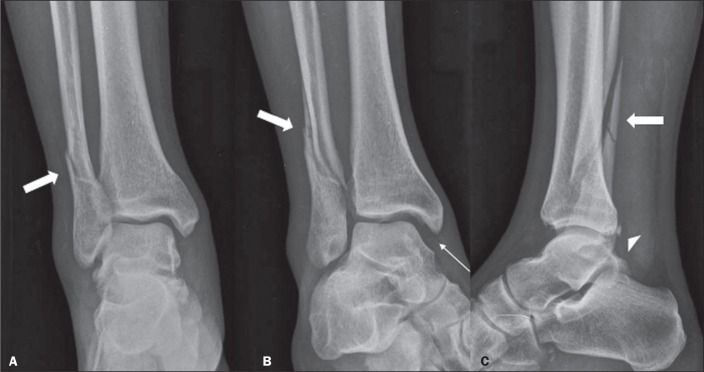
A 45-year-old man who had sprained his right ankle while skateboarding. Anteroposterior, oblique, and lateral X-rays of the right ankle (A, B, and C, respectively) showing a trans-syndesmotic supination fracture of the ankle with external rotation, with a trace spiral fracture of the distal fibula (thick arrows). We highlight the increase in the medial clear space in the incidence of mortise (thin arrow), inferring damage to the deltoid ligament and small injury to the posterior malleolus (arrowhead).

Ankle fractures are common, occurring mainly in inversion or eversion injuries. The indications for surgical treatment include loss of joint congruence, displaced fracture of the medial malleolus, fracture of the lateral malleolus with shortening or displacement, bimalleolar fracture, and open fractures. Attention should be paid to injury of the tibiofibular syndesmosis (Figure 11), which is characterized by a reduction in the tibiofibular overlap on anteroposterior and mortise X-rays^(1)^.

### Lisfranc fracture-dislocation

**Figure 12:**
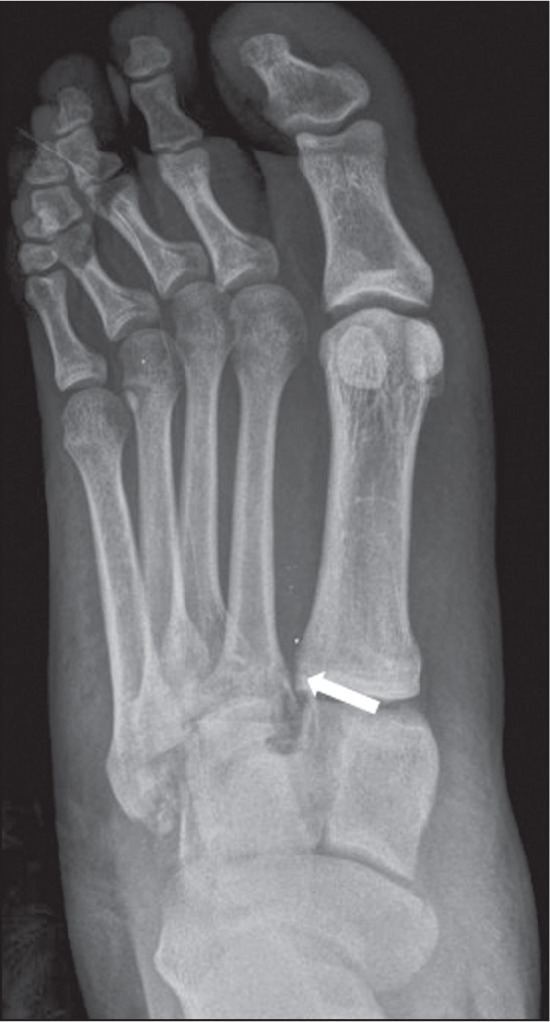
A 27-year-old woman, victim of a motorcycle versus car collision, who evolved to pain and edema in the right foot. Anteroposterior X-ray of the right foot showing a homolateral Lisfranc fracture-dislocation. Note the increase in the distance between the first and second metatarsals (arrow), which is diagnostic of a Lisfranc injury.

Lisfranc ligament injury is characterized by traumatic disjunction of the medial cuneiform joint and the base of the second metatarsal. Lisfranc fracture-dislocation is a partial or complete loss of bony and ligamentous stability at the level of the tarsometatarsal joint (Figure 12). Lisfranc fracture-dislocations are uncommon, accounting for only 0.2% of all fractures. They typically affect men in the third decade of life, arising from indirect rotational forces and axial load with the forefoot flexed, in motor vehicle accidents, falls, and sports activities. Depending on the direction of metatarsal displacement, Lisfranc fracture-dislocations are subdivided into three types: ipsilateral, characterized by lateral displacement of the first to fifth metatarsals or lateral displacement of the second to fifth metatarsals, with persistence of joint congruence of the first metatarsal; divergent, characterized by lateral displacement of the second to fifth metatarsals and medial displacement of the first metatarsal; and isolated, characterized by dorsal displacement of only a few metatarsals. The main imaging finding is misalignment of the second tarsometatarsal joint, characterized by lateral displacement of the base of the second metatarsal in an anteroposterior view, with or without vertical misalignment in a lateral view^(1,10)^.

The findings, classifications, and indications for the surgical management of the fractures of the lower limbs described in this essay are summarized in Table 2.

**Table 2:** Lower limb fractures and their respective mechanisms of trauma, findings that indicate surgical treatment, and the main surgical treatment adopted.

Fracture	Segment	Mechanism of trauma	Findings that indicate surgical treatment	Main surgical treatment adopted
Pelvic ring (open book)	Pelvis	High energy blunt trauma	Diastasis of the pubic symphysis > 25.0 mm; displacement of the sacroiliac joint; posterior (sacral or sacroiliac) injury, together with anterior injury (fracture of the branches or opening of the pubic symphysis) – except for type 1 lateral shear	External fixation; open reduction with internal fixation
Intertrochanteric	Proximal femur	Elderly: falls; young people: high-energy traumas	Any, except the following: clinical conditions that contraindicate surgery; isolated fracture of the greater or lesser trochanter	Open reduction with internal fixation; closed reduction with internal fixation
Femoral neck	Proximal femur	Elderly: falls; young people: high-energy traumas	Any, although surgery can be conservative if the following are present: fractures without displacement or impacted in valgus (controversial); clinical conditions that contraindicate surgery	Internal fixation; arthroplasty
Tibial plateau	Proximal tibia	Trauma with axial force vector	Joint depression > 2 mm (including depression or shear); change of limb axis (varus or valgus); enlargement of the plateau (condylar diastasis > 5 mm); deviated fractures	Open reduction with internal fixation; external fixation
Ankle	Tibia and distal fibula	Ankle sprain	Loss of joint congruence; malleolar fracture with deviation > 2.0 mm; lateral malleolar fracture with ligament injury: deltoid ligament injury—medial clear space > 4.0 mm; syndesmotic injury—tibiofibular overlap < 10.0 mm in anteroposterior view and < 1.0 mm in true anteroposterior/mortise view; Bimalleolar or trimalleolar fracture	Open reduction with internal fixation
Tarsometatarsal (Lisfranc)	Tarsometatarsal	Motor vehicle accidents, crushes, falls from a height, and sports injury	Deviation > 1.0 mm; joint incongruity > 2.0 mm; instability or diastasis on a weight-bearing X-ray; compartment syndrome; exposed fracture	Open reduction with internal fixation; closed reduction and percutaneous fixation

## CONCLUSION

Fractures are common findings in emergency radiology practice. Therefore, it is important for radiologists to be familiar with the main mechanisms of trauma and the imaging findings that inform orthopedist decisions regarding the appropriate surgical approach.
